# Early screening of post‐stroke fall risk: A simultaneous multimodal fNIRs‐EMG study

**DOI:** 10.1111/cns.70041

**Published:** 2024-09-24

**Authors:** Zheng Yang, Liu Ye, Lining Yang, Qiuyi Lu, Anqi Yu, Dingqun Bai

**Affiliations:** ^1^ Department of Rehabilitation Medicine The First Affiliated Hospital of Chongqing Medical University Chongqing China

**Keywords:** diagnostic efficacy, functional near‐infrared spectroscopy, post‐stroke fall risk, stroke

## Abstract

**Background:**

Stroke is the third‐leading cause of death and disability, and poststroke falls (PSF) are common at all stages after stroke and could even lead to injuries or death. Brain information from functional near‐infrared spectroscopy (fNIRs) may precede conventional imaging and clinical symptoms but has not been systematically considered in PSF risk prediction. This study investigated the difference in brain activation between stroke patients and healthy subjects, and this study was aimed to explore fNIRs biomarkers for early screening of PSF risk by comparing the brain activation in patients at and not at PSF risk.

**Methods:**

In this study, we explored the differences in brain activation and connectivity between stroke and healthy subjects by synchronizing the detection of fNIRs and EMG tests during simple (usual sit‐to‐stand) and difficult tasks (sit‐to‐stand based on EMG feedback). Thereby further screened for neuroimaging biomarkers for early prediction of PSF risk by comparing brain activation variability in poststroke patients at and not at fall risk during simple and difficult tasks. The area under the ROC curve (AUROC), sensitivity, and specificity were used to compare the diagnostic effect.

**Results:**

A total of 40 patients (22 not at and 18 at PSF risk) and 38 healthy subjects were enrolled. As the difficulty of standing task increased, stroke patients compared with healthy subjects further increased the activation of the unaffected side of supplementary motor area (H‐SMA) and dorsolateral prefrontal cortex‐Brodmann area 46 (H‐DLFC‐BA46) but were unable to increase functional connectivity (Group*Task: *p* < 0.05). More importantly, the novel finding showed that hyperactivation of the H‐SMA during a simple standing task was a valid fNIRs predictor of PSF risk [AUROC 0.74, *p* = 0.010, sensitivity 77.8%, specificity 63.6%].

**Conclusions:**

This study provided novel evidence that fNIR‐derived biomarkers could early predict PSF risk that can facilitate the widespread use of real‐time assessment tools in early screening and rehabilitation. Meanwhile, this study demonstrated that the higher brain activation and inability to increase the brain functional connectivity in stroke patients during difficult task indicated the inefficient use of brain resources.

## Graphical abstract

This study provided novel evidence that fNIR‐derived biomarkers could early predict PSF risk that can be widely used in clinical practice. Meanwhile, this study demonstrated that the higher brain activation and inability to increase the brain functional connectivity in stroke patients during difficult task indicated the inefficient use of brain resources.

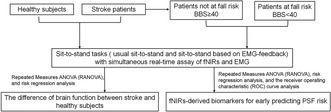

## 1 INTRODUCTION

Stroke is the third‐leading cause of death and disability^1^ resulting in up to 50% of survivors being chronically disabled.^2^ Falls are common at all stages after stroke and the consequences include functional limitations, minor or serious injuries, and even death.^3^ Currently, clinical screening post‐stroke fall (PSF) risk relies on scales, and clinical presentations assessed by scales are later than changes in brain function.^4^ These pose a major obstacle to accomplish rapid and early screening for PSF risk. Therefore, the development of early and quantitative screening tests for PSF risk is essential to prescribe targeted prevention interventions.

Functional near‐infrared spectroscopy (fNIRs) as a noninvasive brain functional neuroimaging technique has great potential to be an early quantitative screening tool,^5^ as it is based on neurovascular coupling (NVC) and allows real‐time detection reflecting the hemodynamic changes during different tasks that may precede the appearance of traditional imaging and clinical symptoms.^6^, ^7^ Sit‐to‐stand is a necessary prerequisite for walking, an important early indicator of balance control and independence in activities of daily living. Meanwhile, sit‐to‐stand is an important training for balance function, and sit‐to‐stand test is commonly used in clinical settings to assess fall risk.^8^

fNIRs has been increasingly applied to screen neurological disorders^5^, ^9^, ^10^, ^11^, ^12^ and poststroke dysfunction.^5^, ^9^, ^10^ Few studies^13^, ^14^ explored the relationship between fNIRs‐derived information with balance function. The use of maintenance standing postural perturbation or walking paradigm in several studies^15^, ^16^, ^17^, ^18^ required higher function and lacked certain safety thus resulting in certain limitations for early screening in patients with poor function. Previous studies have shown that heightened electromyography (EMG) amplitude in the affected rectus femoris in the perturbation paradigm was associated with post‐stroke falls.^19^ However, only a few fNIRs studies^20^, ^21^ recorded synchronous EMG or strength change so that it failed to elucidate the relationship of muscle change with cortical activation in patients.

Therefore, to address these concerns, this study investigated the difference in brain activation between stroke patients and healthy subjects, and this study explored neuroimaging biomarkers to early predict PSF risk by comparing the brain activation in patients at and not at PSF risk during simple task (usual sit‐to‐stand) and difficult task (sit‐to‐stand based on EMG feedback).

## 2 METHOD

### 2.1 Study design

In this cross‐sectional study, all subjects received simultaneous detection of fNIRs and EMG. This study investigated the difference in brain activation between stroke patients and healthy subjects when they completed different sit‐to‐stand tasks, and further screened the fNIRs‐derived poststroke fall risk biomarkers by comparing stroke patients at or not at fall risk during different tasks.

### 2.2 Participants

A total of 82 subjects were informed in this study (43 stroke patients and 39 healthy subjects), of whom four withdrew from the trial, including three patients and one healthy subject who refused during the trial. Participants were recruited from the Department of Rehabilitation Medicine, the First Affiliated Hospital of Chongqing Medical University, Chongqing, China.

General inclusion criteria for stroke and healthy subjects included^1^ aged 18–85 years^2^; able to complete the sit‐to‐stand transition independently or with minor assistance^3^; able to follow instructions to complete the trial, and^4^ capable of providing informed consent for the study. Stroke subjects were included if they were diagnosed with stroke confirmed by neuroimaging (CT or MRI) evaluation.

The exclusion criteria for stroke and healthy subjects were as follows^1^: inability to tolerate the test,^2^ pregnant or lactating women,^3^ unrepaired cranial flaps after cranial surgery or having metal implants, and^4^ history of serious psychiatric comorbidity, other neurological disorders, acute cardiopulmonary dysfunction, multi‐organ failure, brain tumors, and seizures.

### 2.3 Standard protocol approvals, registrations, and patient consents

The study was approved by the Medical Research Ethics Committee of The First Affiliated Hospital of Chongqing Medical University and was performed according to the principles of the Declaration of Helsinki. All participants were informed consent before participation. This study was registered with ClinicalTrials.gov (NCT06062407).

### 2.4 Evaluation of poststroke fall risk

Stroke patients were divided into two groups according to the Berg Balance Scale (BBS) assessment of balance level.^22^ Stroke group 1 (ST1): stroke patients not at fall risk whose BBS score is higher than or equal to 40; Stroke group 2 (ST2): stroke patients at fall risk whose BBS score less than 40.

### 2.5 Procedures

All participant characteristics (age, sex, education, exercise habit, comorbidities including diabetes, hypertension, and hyperlipidemia), as well as the stroke history information (stroke type, stroke onset, and affected hemisphere), National Institutes of Health Stroke Scale (NIHSS), and Minimum Mental State Examination (MMSE) of patients were obtained.^23^

The sit‐to‐stand paradigm required subjects to sit on a 47 cm‐high chair, feet hip‐width apart and toes under knees, stand up, and then sit down.^24^ All subjects were asked to perform sit‐to‐stand tasks in 2 conditions^1^: simple task: usual sit‐to‐stand without EMG feedback, and^2^ difficult task: sit‐to‐stand based on EMG feedback. Before the difficult task, rehabilitation therapists instructed sit‐to‐stand with EMG feedback to all subjects for 5–10 minutes until they learned to adjust sit‐to‐stand based on EMG feedback. Two physicians (Y.L. and Y.Z.) performed an examination, blinded to the BBS score and medical history of patients during the process of examination.

### 2.6 Functional near‐infrared spectroscopy measurement

#### 2.6.1 fNIRs task paradigm

**FIGURE 1:**
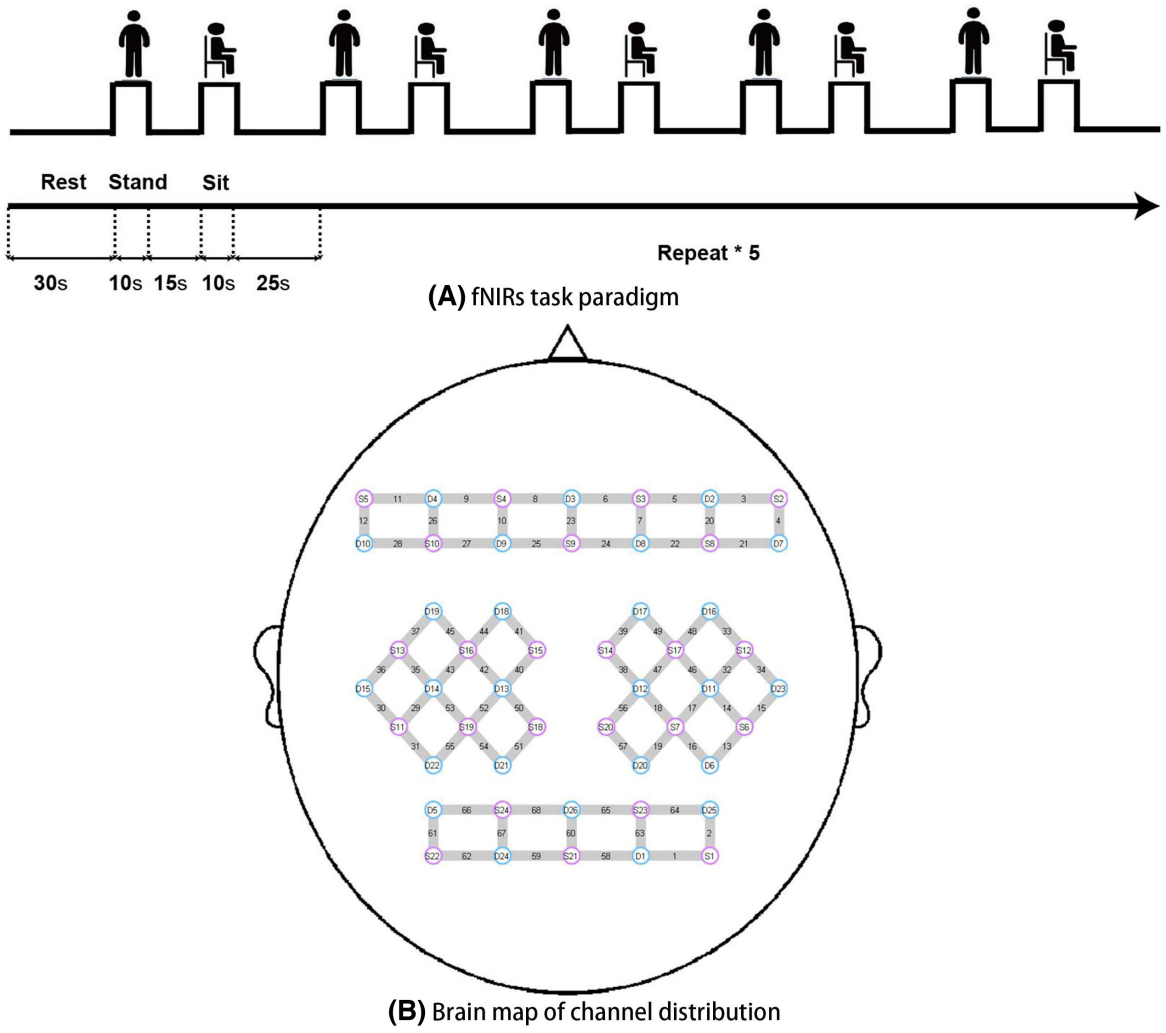
Acquisition of fNIRs. (A) fNIRs task paradigm. The paradigm consisted of a 10s standing task followed by a 15 s rest, a 10s sitting task followed by a 15 s rest, and a 10s rest, repeated five times. (B) Brain map of channel distribution. A total of 68 channels were built by 24 light sources and 26 detectors. The brain areas mainly observed in this study were Primary Motor Cortex (M1, Brodmann area (BA) 4: S7‐D12, S18‐D13, S19‐D13, S20‐D12), Supplementary Motor Area (SMA, BA6: S12‐D11, S13‐D14, S14‐D12, S14‐D17, S15‐D13, S15‐D18, S16‐D13, S16‐D14, S16‐D18, S17‐D11, S17‐D12, S17‐D17), and Dorsolateral Prefrontal Cortex (DLFC, BA9: S12‐D16, S13‐D19, S16‐D19, S17‐D16 and BA46: S2‐D2, S5‐D4, S8‐D2, S8‐D8, S10‐D4, S10‐D9).

Figure 1A illustrates fNIRs task paradigm. Prior to the experiment, participants were asked to take a 30s rest in a seated position. The paradigm consisted of a 10s standing task followed by a 15 s rest, a 10s sitting task followed by a 15 s rest, and 10s rest, repeated five times.^25^ All subjects and testers were not allowed to talk or communicate to avoid interference.

#### 2.6.2 Acquisition of fNIRs

Details of fNIRS data acquisition have been described previously.^26^, ^27^ Briefly, NirScan‐6000A equipment (Danyang Huichuang Medical Equipment Co., Ltd., China) was used to continuously measure the concentration changes in brain oxygenated hemoglobin considered as cortical activation changes. The near‐infrared light was conveyed at wavelengths of 730 nm, 808 nm, and 850 nm, with a sample rate of 11 Hz. Figure 1B shows a total of 68 channels were built by 24 light sources and 26 detectors for fNIRs measurement. The cortical region of interest (ROI) mainly observed in this study were primary motor cortex (M1: Brodmann area (BA) 4), supplementary motor area (SMA: BA6), and dorsolateral prefrontal cortex (DLFC: BA9 and BA46). Imaging data from patients with left‐sided lesions were flipped horizontally before data analysis, thus the affected hemisphere being the right hemisphere.^28^

#### 2.6.3 Pre‐processing and analysis of fNIRs data

The fNIRS signals were preprocessed and analyzed using NirSpark software (Danyang Huichuang Medical Equipment Co., Ltd., China).^29^, ^30^ (1) Measurement data with poor‐quality signals were rejected, (2) Motion artifacts in channels were amended by spline interpolation algorithm.^29^ Any signal change beyond 5 standard deviations (std_thr >6) and 0.5 amplitude (amp_thr >0.5) of the entire time series was considered a motion artifact for tighter control of data quality,^31^ and (3) Then, the raw data were band‐pass filtered between 0.01 and 0.2 Hz to remove physiological noise (e.g., respiration, cardiac activity, and low‐frequency signal drift). The filtered signals were converted to relative concentration changes of HbO and HbR based on the modified Beer–Lambert law^26^ (Specific formulas were in the supplementary materials‐method—Data S1). The hemodynamic response function (HRF) was set to an initial time of 0 s and an end time of 10 s, with 2 s before the initial time as the reserved baseline state and 10 s as task time for a block paradigm, for a total of 5 block paradigms. The oxyhemoglobin concentrations for each block paradigm were superimposed and averaged to generate block average results. Functional connectivity (FC) matrix calculated by Pearson correlation analyses with 5‐min (from the first standing to the last sitting) between each pair of channels from ROIs.^26^, ^27^ The average of correlation coefficients (r) was used as a general FC value for group comparison.^29^

### 2.7 EMG Measurement

The activation of bilateral rectus femoris (RF) and biceps femoris (BF) during simple and difficult sit‐to‐stand tasks were synchronously detected by a wireless EMG system with signals recorded at 1000 Hz (BTS Bioengineering Corp, Italy).^32^ Details of EMG detection have been described previously^33^, ^34^ (Specific formulas and processing were in the supplementary materials‐method—Data S1).

### 2.8 Statistical analyses

The normal distribution of data was evaluated with Kolmogorov–Smirnov tests. The t‐test was used for comparison of normally distributed data, the Mann–Whitney U test for abnormally distributed data, respectively, and the Chi‐Square test for categorical variables. RANOVA to determine different interactions (task × group) of fNIRs‐derived HbO_2_ was used to screen for statistically significant ROIs, and the RANOVA was further completed on the FC of these significant ROIs. The data were corrected by the false discovery rate (FDR). Then, multivariate risk regression analysis was conducted to explore the relationship between these screened fNIRs‐derived biomarkers and stroke or PSF risk. Based on the significant fNIRs‐derived biomarkers in multivariate risk regression, diagnostic efficiency for fall risk after stroke was assessed by the ROC curve analysis including area under the curve (AUC), sensitivity, and specificity. The optimal cut point value is the maximum value of the Youden index (Youden index = Sensitivity + Specificity – 1). The software SPSS version 27.0 (IBM Corp) was used for all statistical analyses.

## 3 RESULTS

### 3.1 Participants characteristics

**FIGURE 2:**
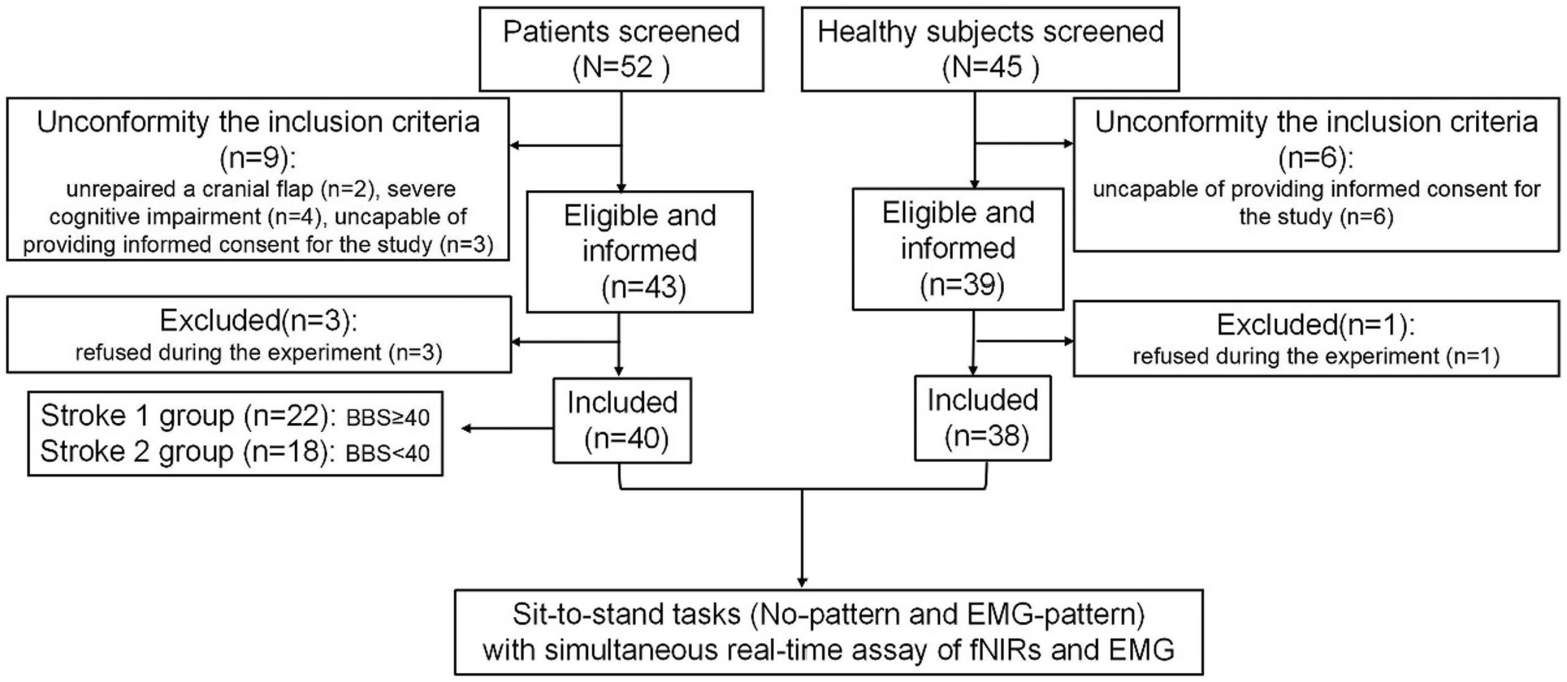
The flow diagram. A total of 82 subjects were informed in this study (43 stroke patients and 39 healthy subjects), of whom four withdrew from the trial, including three patients and one healthy subjects who refused during the trial.

Figure 2 is the flow diagram. The final data consisted of 40 patients [stroke group 1 (ST1): 22 patients not at fall risk, stroke group 2 (ST2): 18 patients at fall risk] and 38 healthy subjects. There were no statistically significant differences in age, sex, years of education, or exercise habits between stroke and healthy subjects, as well as ST1 and ST2. Among stroke patients, there was similarly no significant difference in types of stroke, stroke onset, or affected hemisphere between the ST1 and ST2 groups (Table 1). There were no adverse events during the trial. The following results were the fNIRs outcomes, and Table S1 shows the comparison of EMG outcomes indicating that the increased muscle activation from simple tasks to difficult tasks in stroke patients.

**TABLE 1:** Demographics and clinical characteristics.

Characteristic	HC (*n* = 38)	ST (*n* = 40)	*p*_*1*_	ST1 (*n* = 22)	ST2 (*n* = 18)	*p*_*2*_
Age (years, mean ± SD)	54.29 ± 14.68	55.70 ± 12.99	0.654	53.00 ± 14.63	59.00 ± 10.08	0.148
Sex Male, *n* (%)	29 (52.6%)	31 (52.5%)	0.901	18 (81.8%)	13 (72.2%)	0.625
Years of education (years, mean ± SD)	10.57 ± 3.75	11.65 ± 3.72	0.208	12.45 ± 3.46	10.67 ± 3.88	0.132
Exercise habit Yes, *n* (%)	9 (23.7%)	28 (30.0%)	0.530	8 (36.4%)	4 (22.2%)	0.332
Ischemic Hemorrhagic, *n* (%)	‐	15 (37.5%)	‐	6 (27.3%)	9 (50.0%)	0.140
Stroke onset (months, mean ± SD)	‐	7.65 ± 6.87	‐	8.00 ± 8.52	7.22 ± 4.29	0.727
Affected hemisphere Right, *n* (%)	‐	22 (55.0%)	‐	14 (63.6%)	8 (44.4%)	0.225
MMSE	‐	24.10 ± 3.73	‐	24.64 ± 3.72	23.41 ± 3.73	0.32

*Note*: *p*_*1*_ indicated that the *p*‐value of stroke subjects compared with healthy subjects. *p*_*2*_ indicated that the *p* value of stroke subjects at being fall risk compared with stroke subjects not at being fall risk.

Abbreviations: HC, healthy subjects; ST, stroke patients; ST1, stroke patients not at fall risk; ST2, stroke patients at fall risk.

### 3.2 RANOVA analysis of fNIRs outcomes between stroke and healthy subjects during tasks

**FIGURE 3:**
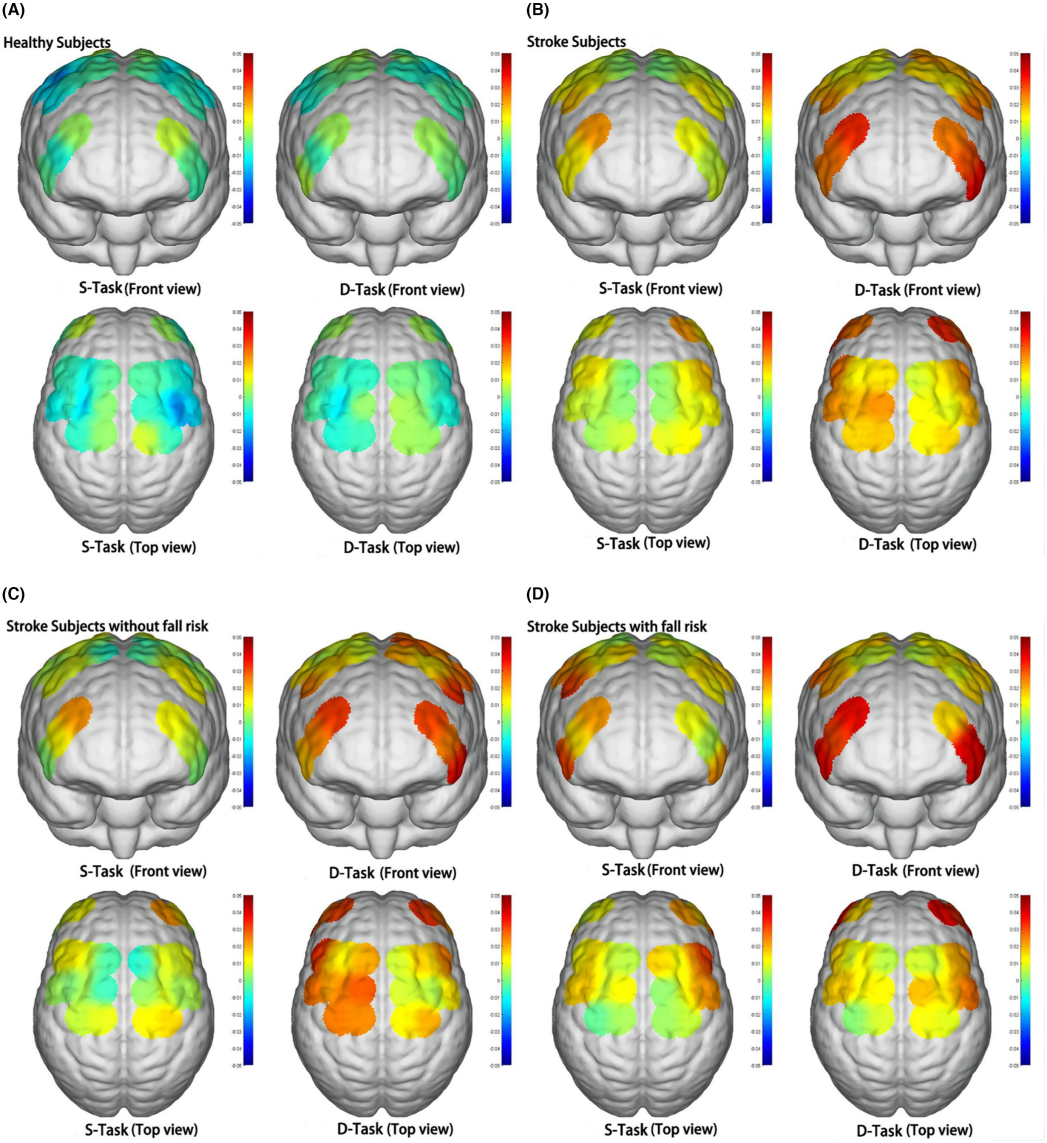
The Brain map of Changes in mean HbO_2_ in the aimed ROI between groups during tasks. (A) Changes in mean level of HbO_2_ in the aimed ROI in healthy subjects during simple and difficult standing tasks in front and top views. (B) Changes in mean level of HbO_2_ in the aimed ROI in stroke subjects during simple and difficult standing tasks in front and top views. (C) Changes in mean level of HbO_2_ in the aimed ROI in stroke subjects without fall risk during simple and difficult standing tasks in front and top views. (D) Changes in mean level of HbO_2_ in the aimed ROI in stroke subjects with fall risk during simple and difficult standing tasks in front and top views. D‐Task, Difficult task; S‐Task, Simple task.

**FIGURE 4:**
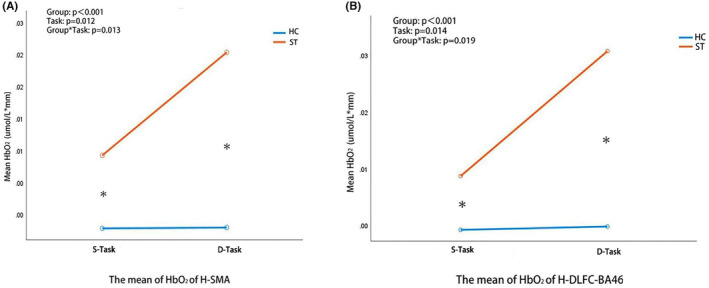
RANOVA Analysis of *fNIRs outcomes* between stroke and healthy subjects during tasks. (A) RANOVA Analysis of fNIRs‐derived HbO_2_ in the unaffected of SMA between stroke and healthy subjects during simple and difficult standing tasks. (B) RANOVA Analysis of fNIRs‐derived HbO_2_ in the unaffected of DLFC‐BA46 between stroke and healthy subjects during simple and difficult standing tasks. D‐Task, Difficult task; S‐Task, Simple task.

RANOVA analysis of each ROI [the unaffected and affected side of M1(BA4)/SMA(BA6)/DLFC(BA9)/DLFC(BA46)] between stroke and healthy groups during two tasks was conducted. The simple task was usual sit‐to‐stand without EMG feedback, and difficult task was sit‐to‐stand based on EMG feedback. As the difficulty of task increased from simple to difficult standing task, the HbO_2_ of the unaffected side of SMA and DLFC‐BA46 (H‐SMA and H‐DLFC‐BA46) increased in stroke patients but remained unchanged in healthy subjects, (Figures 3A,B and 4A,B, Group*Task: H‐SMA *p* = 0.013, H‐DLFC‐BA46 *p* = 0.019). Compared with healthy subjects, stroke patients had higher HbO_2_ in simple and difficult standing tasks (simple task: H‐SMA *Z* = −2.66, *p* = 0.016, H‐DLFC *Z* = −1.99, *p* = 0.047; difficult task: H‐SMA *Z* = −3.43, *p* = 0.001, H‐DLFC *Z* = −3.86, *p* < 0.001). Comparison analysis of remaining brain regions in standing task and all brain regions in sitting task didn't show a significant difference (Group*Task: *p* > 0.05).

According to the above screened brain regions (the unaffected side of SMA and DLFC‐BA46), the FC analysis of SMA‐self, DLFC(BA46)‐self, and SMA‐DLFC(BA46) was completed. Stroke patients had decreased connectivity tended of SMA‐self, DLFC(BA46)‐self, and SMA‐DLFC(BA46) when completing difficult task compared with simple task, whereas the brain connectivity increased in the healthy subjects [Figure S1A–C, Group*Task: SMA‐self *p* = 0.019, DLFC(BA46)‐self *p* = 0.013, SMA‐DLFC(BA46) *p* = 0.010]. Compared with healthy subjects, stroke patients had higher FC of DLFC‐self, and SMA‐DLFC(BA46) in simple standing task [SMA‐self *t* = −1.64, *p* = 0.105, DLFC(BA46)‐self *t* = −2.73, *p* = 0.024, SMA‐DLFC(BA46) *t* = −2.69, *p* = 0.014].

### 3.3 RANOVA analysis of fNIRs outcomes between stroke patients at and not at fall risk during tasks

**FIGURE 5:**
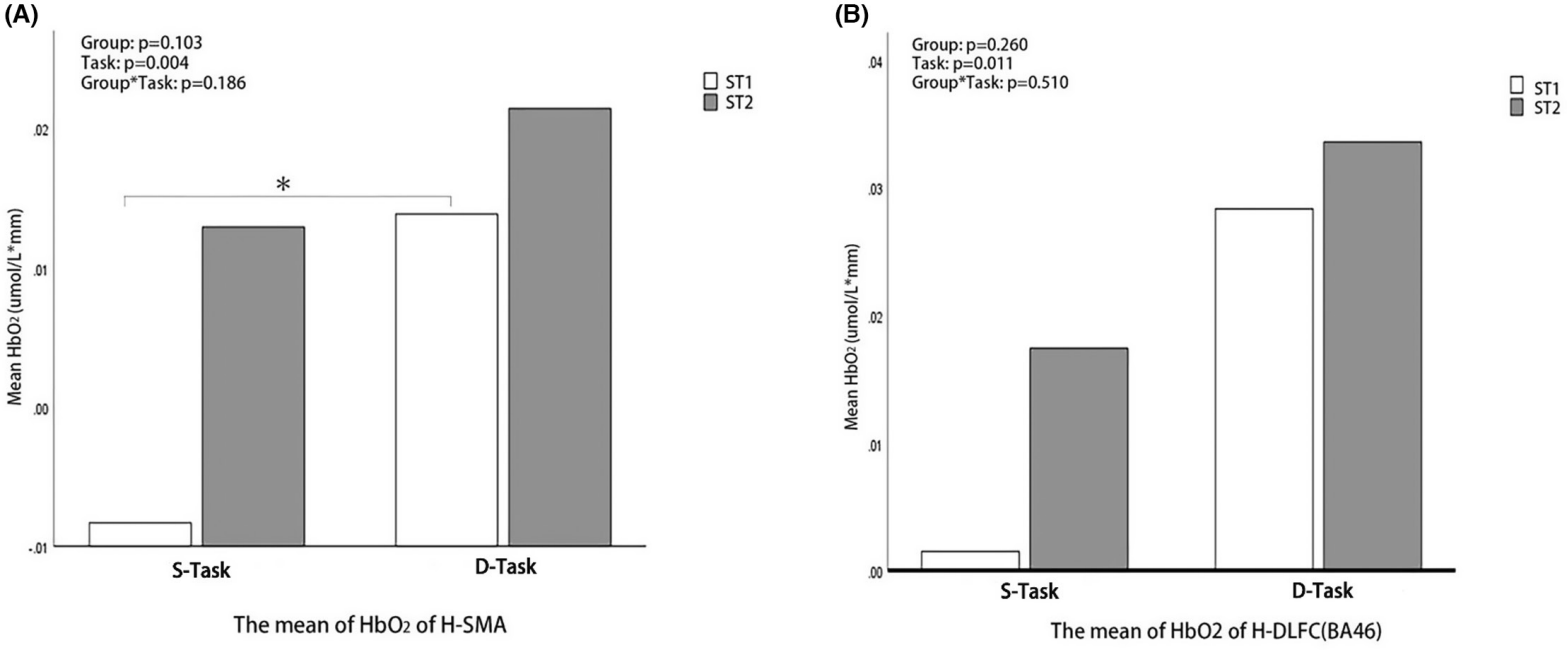
RANOVA Analysis of fNIRs outcomes between stroke patients at and not at fall risk during tasks. (A) RANOVA Analysis of fNIRs‐derived HbO_2_ in the unaffected of SMA between stroke patients at and not at fall risk during simple and difficult standing tasks. (B) RANOVA Analysis of fNIRs‐derived HbO_2_ in the unaffected of DLFC‐BA46 between stroke patients at and not at fall risk during simple and difficult standing tasks. D‐Task, Difficult task; S‐Task, Simple task.

Comparison analysis of the above‐screened ROI (the unaffected side of SMA and DLFC‐BA46) in the ST1 and ST2 groups was conducted (Figures 3C,D and 5A,B). Comparison of the mean of HbO_2_ from simple to difficult standing task between stroke groups didn't show significant difference (H‐SMA: Group*Task *p* = 0.186, H‐DLFC‐BA46 Group*Task *p* = 0.510). Compared with stroke patients without fall risk, stroke patients at fall risk had significantly higher HbO_2_ of the unaffected side of SMA in simple standing tasks (H‐SMA: *Z* = −2.58 *p* = 0.010, H‐DLFC: *Z* = −1.01 *p* = 0.314). However, none of brain network connections were significantly different when compared in the ST1 and ST2 groups (Group*Task *p* > 0.05) [Figure S2A–C].

### 3.4 Risk regression analysis of stroke and post‐stroke fall risk

To explore the relationship between the HbO_2_/FC of above‐screened brain regions (the unaffected of SMA and DLFC‐BA‐46) and stroke, 2 models of regression analysis were conducted. Model 1 was a single‐factor regression analysis. As comorbid conditions including hypertension, diabetes mellitus, and hyperlipidemia are risk factors for stroke, and the difficult task involves cognitive ability, model 2 adjusted for basic demographic information (age, sex, years of education, whether having combined diseases including hypertension, diabetes, and hyperlipidemia), and fNIRs outcomes with statistical significance in RANOVA analysis (the FC of DLFC‐self, SMA‐self, and DLFC‐SMA in the simple and difficult tasks, and the HbO_2_ of a simple task in each ROI). The regression analyses showed that the mean HbO_2_ of H‐SMA and H‐DLFC‐BA46 in difficult task, respectively, were associated with stroke (Model 1: H‐SMA *p* = 0.001, H‐DLFC‐BA46 *p* < 0.001; Model 2: H‐SMA *p* = 0.013, H‐DLFC‐BA46 *p* = 0.015) (Table 2), but the mean HbO_2_ of both brain regions in simple task showed no statistically significant after the adjusting of model 2 (*p* > 0.05).

**TABLE 2:** Regression analysis of stroke and post‐stroke fall risk.

	Model 1		Model 2	
	Exp (β) 95% CI	*p*	Exp (β) 95% CI	*p*
*Stroke*				
SMA				
Difficult task	1.039 (1.015–1.063)	0.001	1.052 (1.011–1.094)	0.013
DLFC‐BA46				
Difficult task	1.049 (1.020–1.078)	<0.001	1.071 (1.013–1.132)	0.015
*Poststroke fall risk*				
SMA				
Simple task	1.039 (1.003–1.076)	0.031	1.110 (1.016–1.213)	0.021

*Note*: Regression Analysis of Stoke: Model 1 had no correction factor. Model 2 adjusted for basic demographic information (age, sex, years of education, whether having combined diseases including hypertension, diabetes, and hyperlipidemia) and fNIRs outcomes (the FC of DLFC‐self, SMA‐self and DLFC‐SMA in the simple and difficult tasks, and the HbO_2_ of simple task in each ROI) based on the model 1. Regression Analysis of Poststroke Fall Risk: Model 1 had no correction factor. Model 2 adjusted for age, sex, whether having combined diseases inculding hypertension, diabetes, and hyperlipidemia, history of stroke (stroke type, stroke duration, cerebral hemorrhage site), MMSE, and NIHSS.

As the difference analysis only showed that the HbO_2_ of H‐SMA in simple task was statistically significant, the relationship between the HbO_2_ of H‐SMA and poststroke fall risk was explored. Model 1 was a single‐factor regression analysis. Stroke history information, neurological function, and cognitive function were associated with the fall risk after stroke, therefore, model 2 adjusted for age, sex, whether having combined diseases including hypertension, diabetes, and hyperlipidemia, history information of stroke (stroke type, stroke duration, cerebral hemorrhage site), and NIHSS, and MMSE. Regression analyses showed that the mean HbO_2_ of SMA in simple task were associated with poststroke fall risk (Model 1: H‐SMA *p* = 0.031, Model 2: H‐SMA *p* = 0.021) (Table 2).

### 3.5 ROC analysis of post‐stroke fall risk

**FIGURE 6:**
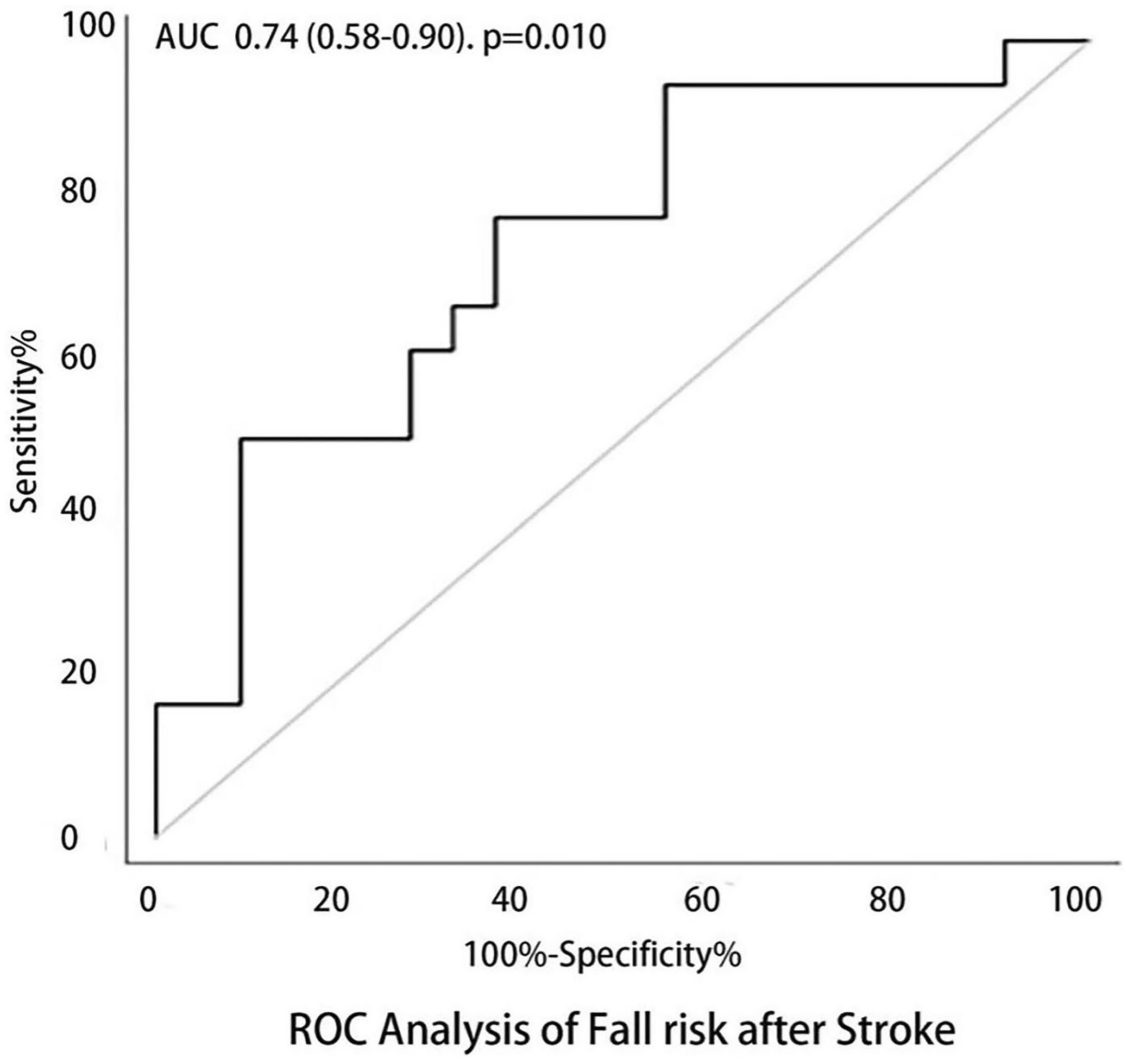
ROC Analysis of Post‐Stroke Fall Risk. The ROC Analysis of Post‐Stroke Fall Risk: AUROC 0.74 (0.58–0.90), *p* = 0.010, sensitivity 77.8%, specificity 63%.

As regression analysis showed the mean HbO_2_ of unaffected side of SMA in the simple task significantly associated with poststroke fall risk, we used the mean HbO_2_ of H‐SMA in the simple task for the ROC analysis. The AUC of the area under the ROC curve for the fall risk after stroke was 0.74 (95%CI 0.58–0.90, sensitivity 77.8% specificity 63.6%, *p* = 0.010) (Figure 6).

## 4 DISCUSSION

The emerging evidence suggested the necessary and urgency of the development of neuroimaging biomarkers for early objective assessment.^18^ In this study, the difference analysis showed that compared with healthy subjects, stroke patients had higher activation but were unable to increase the brain functional connectivity in difficult task performance indicating inefficient use of brain resources. Moreover, as a real‐time synchronized of fNIRs and EMG experiment, this study ultimately found that fNIRs‐derived HbO_2_ indicators with high diagnostic effect for PSF risk, which provided a rapid and simple initial screening test as preliminary assessment for early recognition of PSF to prescribe targeted prevention interventions.

### 4.1 The difference in brain activation and network connectivity between stroke and healthy subjects

This study found that the cortex activation of stroke patients had increased during both simple and difficult standing tasks, and further increased as the difficulty of the task increased. Meanwhile, stroke patients had higher FC in simple task compared to healthy subjects. However, healthy subjects but not stroke patients could increase effective connectivity in difficult paradigms. In contrast, the FC analysis showed no difference in stroke patients being at or not at fall risk.

A clinical^35^ study showed that stroke survivors exhibited higher levels of prefrontal activation during simple and difficult walking tasks compared to healthy controls. A meta‐analysis^36^ also provided evidence that stroke patients also had a further increase in prefrontal activation during difficult‐task walking compared with standing or simple‐task walking. These studies confirmed that stroke patients with more excessive activation indicated inefficient use of brain resources to complete more difficult task. Meanwhile, the present study found that the relevant brain regions were SMA and DLFC‐BA46. Previous studies mainly focused on prefrontal cortex. However, accumulating evidence suggests that SMA and DLFC play an important role in postural balance control and a key region for balance recovery after hemiplegic stroke.^17^, ^37^

Stroke patients had higher FC during a simple task, which may be related to the need for patients to recruit a wider range of brain regions to perform the same usual motor task. Neurological disconnections of stroke patients may lead to functional reorganization of brain networks.^7^, ^38^ Previous fNIRs studies^39^, ^40^ showed an increased clustering coefficient of small‐world properties or local efficiency of network in stroke compared to healthy subjects. In our study, healthy subjects rather than stroke patients were able to increase effective connectivity in difficult task. Another study^28^ also reported that healthy controls but not patients showed higher Prefrontal cortical (PFC) variability in difficult task, which suggested that higher adaptive patterns in brain function were required to maintain successful performance in more complex task. Stroke patients had significantly reduced brain network controllability^41^ and couldn't increase the integration of brain networks and transmission of information in more complex tasks.

### 4.2 Preliminary screening of fNIRs biomarker for the fall risk of post‐stroke

The novel findings suggested that the high activation of the unaffected side of SMA in a simple standing task can be used as an imaging indicator of PSF risk. Compared with patients not at PSF risk, patients at PSF risk had increased brain activation in simple standing task, but the more difficult task could not further increase brain activation.

A study^42^ showed that stroke patients with poorer balance function had increased prefrontal activation. Meanwhile, patients with neurological disorders also exhibited increased activation predisposing an increased fall risk.^36^ Hyperactivation of brain regions is a very promising imaging biomarker for predicting fall risk. Joe Verghese.et al. reported that higher activation levels of PFC during a difficult walking task predicted falls in older adults.^6^ However, our study found that high activation in simple standing task rather than difficult task can be used as a screening indicator for PSF risk. This may be related to a “ceiling” effect^43^ that usual sit‐to‐stand task was already challenging for patients at PSF risk, whereas Joe Verghese studied at healthy subjects whose brain functions were better than stroke patients. Also, this “ceiling” effect resulted that there was no significant difference in the comparison of the change in HbO_2_ from simple to difficult standing task between stroke groups (Group*task: *p* > 0.05, Figure 5A,B). This was confirmed by a study^43^ reporting that the changes of HbO_2_ in the PFC were independent of cognitive load while walking due to usual walking already challenging for stroke patients. At the same time, we screened fNIRs biomarkers by correcting for the MMSE scores in the multifactorial regression analysis (Table 2), and the results showed that fNIRs biomarker was still significantly associated with post‐stroke fall risk, thus also confirming the stable efficacy of fNIRs biomarker for screening post‐stroke fall risk.

Most of previous studies had smaller sample sizes, lacked comparative analysis between stroke patients with different functional states and healthy subjects,^12^, ^42^ and remained at the level of difference and correlation analysis. Meanwhile, the paradigms of previous studies limited the potential for early screening of PSF risk.^12^, ^13^, ^14^, ^15^, ^16^ A study^13^ extracted the event‐related features during ankle dorsiflexion from EEG and fNIRs to predict balance function, but ankle dorsiflexion cannot fully reflect the overall balance function. Some studies showed that some scale assessments also could predict fall risk such as the NIHSS.^44^ In our study, we found that the model 2 of multifactorial regression analysis (Table 2) also showed that fNIRs biomarker was still significantly associated with post‐stroke fall risk after adjusting the NIHSS scores of patients. Meanwhile, the advantage of fNIRs technology superior to these scale assessments is the ability to detect changes that precede clinical performance, and fNIRs that have a potential in deciphering coordinated movements effectively,^45^ so it is more suitable as an early quantitative prediction tool.

The widespread use of imaging biomarkers in stroke depended on experimental design to infer causality.^18^, ^46^ In our study, regression analysis after adjustment for multiple factors and ROC analysis confirmed fNIRs‐derived indicators had high performance for screening stroke and PSF risk. Our findings showed that fNIRs biomarkers have relatively higher sensitivity and relatively lower specificity for screening for poststroke fall risk, which further implies the reliability of fNIRs as a neuroimaging tool for early initial screening in clinical.^47^ The combined use of fNIRs and EMG as real‐time assessments of brain and muscle activation during target tasks could facilitate the widespread use of real‐time assessment tools in early screening and rehabilitation. Stroke patients showed not only increased brain activation on the unaffected side but also a significant increase in EMG on the affected side from simple to difficult tasks (Table S1). This indicated that patients increased activation in the corresponding brain regions when more muscle units were involved in accomplishing difficult task. This combined use could enhance activation in targeted brain regions and muscles through feedback monitored in real‐time, thus further contributing to the development of effective activation paradigms that have great potential in clinical settings. Meanwhile, by monitoring brain function and muscle response in real‐time in patients with different functions, the difficulty and intensity of the paradigm can be adjusted to provide a personalized formula for better patient rehabilitation.

## 5 LIMITATIONS

There are some limitations in this study. This was a single‐center preliminary study lack of follow‐up. As a cross‐sectional study rather than a prospective study, we used the BBS to categorize groups at or not at PSF risk. Second, because it was inherently more difficult for patients at PSF risk to perform usual sit‐to‐stand task independently or with minor assistance, and fewer patients were able to perform the difficult task, thus only 18 patients at PSF risk in this study.

## 6 CONCLUSION

The present study innovatively using the combination of fNIRs and EMG as real‐time assessments of brain and muscle activation during target tasks, provided novel and robust evidence that high activation on the unaffected side of the SMA during the performance of a simple task can serve as a powerful indicator for early screening for PSF. These findings indicated that fNIRs predictors had the potential to be widely used in the community, in primary care clinics, and even in medical examinations to promote appropriate measures to reduce stroke falls after stroke, which also promoted the widespread use of real‐time assessment tools for early screening and rehabilitation. Meanwhile, the present study demonstrated that stroke patients showed higher brain activation when completing difficult tasks and were unable to increase functional brain connectivity, which indicates inefficient utilization of brain resources.

## AUTHOR CONTRIBUTIONS

All authors were involved in the design and execution of this study. Y.Z. and Y.L. wrote the first and subsequent drafts of the manuscript and provided the tables, figures, and references. Y.Z. performed the data analysis and interpretation. B.D.Q. supervised the trial. All authors edited the manuscript and approved the final draft.

## FUNDING INFORMATION

These studies were supported in part by the National Key Research and Development Program of China, Active health and population aging technology response (2023YFC3604501), Program for Youth Innovation in Future Medicine, Chongqing Medical University (W0076), Chongqing Municipal Education Commission, Innovative Research Group of Universities in Chongqing (CXQT21018), The Fund of 2023 Chongqing Research Innovation Program for Graduate Student in the First Affiliated Hospital of Chongqing Medical University (CYYY‐BSYJSCXXM‐202313), Chongqing Talents Program Innovation and Entrepreneurship Demonstration Team (CQYC202203091113).

## CONFLICT OF INTEREST STATEMENT

The authors declare that they have no conflicts of interest.

## TRIAL REGISTRATION INFORMATION

ClinicalTrials.gov Identifier: NCT06062407.

## Supporting information


Data S1.

## Data Availability

The data that support the findings of this study are available on request from the corresponding author.
