# Supplementary material for: Early screening of post‐stroke fall risk: A simultaneous multimodal fNIRs‐EMG study
Source: CNS Neurosci Ther. 2024 Sep 24;30(9):e70041. doi: 10.1111/cns.70041 (PMC11420627; doi:10.1111/cns.70041)
Supplement: Supplementary file 1 — Data S1. [file CNS-30-e70041-s001.docx]

Supplementary-Method

***Analysis of fNIRs data***

The modified Beer-Lambert law as follows：

$$OD^{\lambda_{i}}=\ln\frac{I_{\mathrm{oi}}}{I_{I}}=\left( \varepsilon_{\mathrm{HbO}}^{\lambda_{i}}C_{\mathrm{HbO}}+\varepsilon_{\mathrm{HbR}}^{\lambda_{i}}C_{\mathrm{HbR}} \right)\times r\times DPF^{\lambda_{i}} i=1,2,3 \left( 1 \right)$$

$$\Delta OD^{\lambda_{i}}=\left( \varepsilon_{\mathrm{HbO}}^{\lambda_{i}}\Delta C_{\mathrm{HbO}}+\varepsilon_{\mathrm{HbR}}^{\lambda_{i}}\Delta C_{\mathrm{HbR}} \right)\times r\times DPF^{\lambda_{i}} i=1,2,3 (2)$$

HbO and HbR can be calculated by the following equation^26^：

$$\left( \begin{aligned} \Delta C_{\mathrm{HbO}} \\ \Delta C_{\mathrm{HbR}} \end{aligned} \right)=\left( \begin{matrix} \varepsilon_{\mathrm{HbO}}^{\lambda_{1}} & \varepsilon_{\mathrm{HbR}}^{\lambda_{1}} \\ \varepsilon_{\mathrm{HbO}}^{\lambda_{2}} & \varepsilon_{\mathrm{HbR}}^{\lambda_{2}} \\ \varepsilon_{\mathrm{HbR}}^{\lambda_{3}} & \varepsilon_{\mathrm{HbO}}^{\lambda_{3}} \end{matrix} \right)^{-1}\left( \begin{aligned} \Delta OD^{\lambda_{1}}/(r\times DPF^{\lambda_{1}}) \\ \Delta OD^{\lambda_{2}}/(r\times DPF^{\lambda_{2}}) \\ \Delta OD^{\lambda_{3}}/(r\times DPF^{\lambda_{3}}) \end{aligned} \right) (3)$$

The variable ℇ is the wavelength-dependent extinction coefficient for each hemoglobin types. The DPF (differential path-length factor) is added to account for the true effective path length between source and detector and r represents the linear distance of the paired probes. ∆OD is the change in light absorption, referred to as delta optical density. ∆CHbO and ∆CHbR represent the relative concentration changes of HbO and HbR respectively.

***Analysis of EMG data***

Normalization tests of EMG were adopted the maximum voluntary isometric contraction (MVC) test measured by using the muscle strength test system (REVO-KN-p11, Ultimate Medical).

The EMG signals in standing and sitting tasks were extracted from muscles as the subject moved from sitting to standing and standing to sitting, respectively, and were full-wave rectified and enveloped with a root mean square (RMS) algorithm with a 50-ms window. The RMS is calculated as：

$$RMS=\sqrt{\frac{1}{n}\sum_{i=1}^{n} e(i)^{2}}$$

Finally, The EMG signals in standing or siting tasks were normalized by MVC value from the peak RMS value in the normalization test to yield relative EMG value. Higher relative EMG value indicated a greater motor unit activation requirement.

Supplementary-Figure1
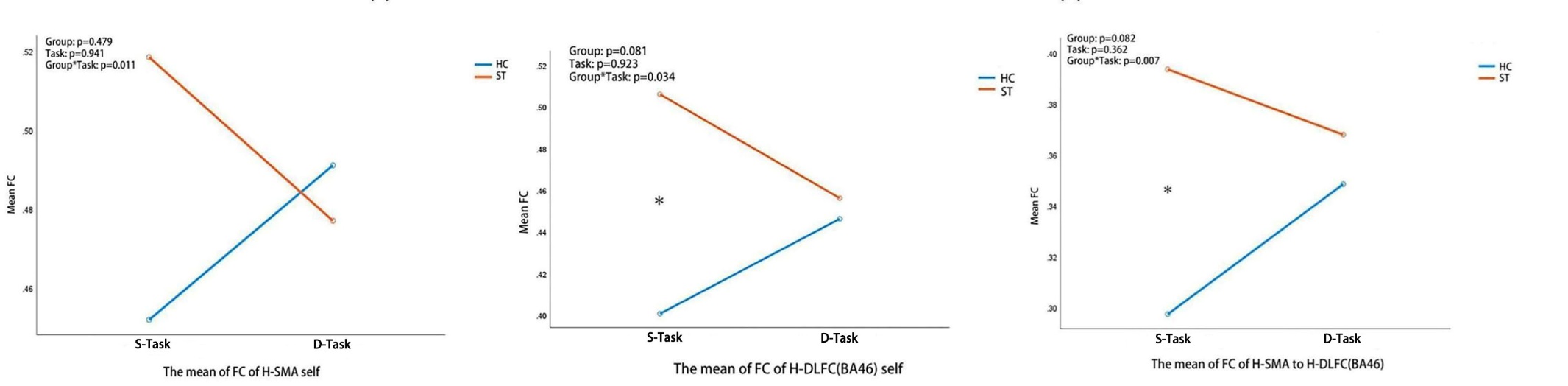


(a) (b) (c)

S-Task: simple task, D-Task: difficult task

Supplementary-Figure1 (a) RANOVA Analysis of fNIRs-derived FC in the unaffected of SMA-self between stroke and healthy subjects during tasks.

Supplementary-Figure1 (b) RANOVA Analysis of fNIRs-derived FC in the unaffected of DLFC(BA46)-self between stroke and healthy subjects during tasks.

Supplementary-Figure1 (c) RANOVA Analysis of fNIRs-derived FC in the unaffected of SMA to DLFC(BA46) between stroke and healthy subjects during tasks.

Supplementary-Figure2


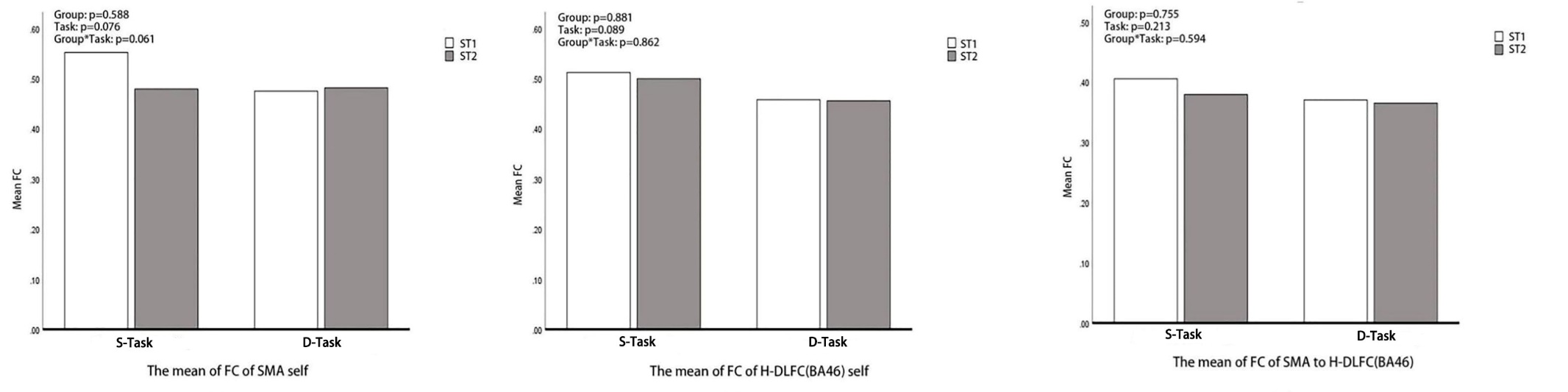


(a) (b) (c)

S-Task: simple task, D-Task: difficult task

Supplementary-Figure2 (a) RANOVA Analysis of fNIRs-derived FC in the unaffected of SMA-self between stroke patients at and not at fall risk during tasks.

Supplementary-Figure2 (b) RANOVA Analysis of fNIRs-derived FC in the unaffected of DLFC(BA46)-self between stroke patients at and not at fall risk during tasks.

Supplementary-Figure2 (c) RANOVA Analysis of fNIRs-derived FC in the unaffected of SMA to DLFC(BA46) between stroke patients at and not at fall risk during tasks.

Supplementary-Table1 Compared with the EMG value between simple and difficult tasks

|  | **HC-Left**  **(t, p)** | **HC-Right**  **(t, p)** | **ST-affected**  **(t, p)** | **ST-unaffected**  **(t, p)** | **ST1-affected**  **(t, p)** | **ST1-unaffected**  **(t, p)** | **ST2-affected**  **(t, p)** | **ST2-unaffected**  **(t, p)** |
| --- | --- | --- | --- | --- | --- | --- | --- | --- |
| **Stand** |  |  |  |  |  |  |  |  |
| **Quadriceps femoris** | 0.002  (p=0.420) | -0.006  (p=0.207) | -0.033  (p＜0.001)* | -0.016  (p=0.193) | -2.528  (p=0.036) * | 0.254  (p=0.801) | -4.073  (p=0.008) * | -1.511  (p=0.155) |
| **Biceps femoris** | -0.003  (p=0.558) | -0.010  (p=0.052) | -0.036  (p＜0.001)* | 0.0099  (p=0.101) | -5.914  (p＜0.001) * | 2.320  (p=0.029) * | -3.763  (p=0.005) * | 0.591  (p=0.565) |
| **Sit** |  |  |  |  |  |  |  |  |
| **Quadriceps femoris** | 0.014  (p=0.224) | -0.003  (p=0.534) | -0.034  (p＜0.001)* | 0.003  (p=0.454) | -6.125  (p＜0.001) * | 0.126  (p=0.901) | -4.017  (p=0.004) * | 1.222  (p=0.243) |
| **Biceps femoris** | -0.004  (p=0.301) | -0.005  (p=0.301) | -0.036  (p＜0.001)* | 0.001  (p=0.774) | -6.41  (p＜0.001) * | -0.52  (p=0.608) | -3.643  (p=0.006) * | 0.69  (p=0.503) |

HC: healthy subjects, ST: stroke patients, ST1: stroke patients not at fall risk, ST2: stroke patients at fall risk.

* indicated the significant significance
